# Identification and validation of DHCR7 as a diagnostic biomarker involved in the proliferation and mitochondrial function of breast cancer

**DOI:** 10.18632/aging.205683

**Published:** 2024-03-22

**Authors:** Yanfeng Wang, Jiaxin Fan, Yongcheng Liu, Jie Du, Boyu Liang, Huxia Wang, Zhangjun Song

**Affiliations:** 1 Department of Surgical Oncology, Shaanxi Provincial People’s Hospital, Shaanxi, China; 2 Department of Clinical Laboratory, Affiliated Hospital of Yan’an University, Shaanxi, China; 3 Department of Health Examination Center, Shaanxi Provincial People’s Hospital, Shaanxi, China; 4 Department of Breast Disease Center, Shaanxi Provincial Tumor Hospital, Shaanxi, China; 5 Department of Geriatric Neurology, Shaanxi Provincial People’s Hospital, Shaanxi, China; 6 Department of Pathology, Affiliated Hospital of Yan’an University, Shaanxi, China

**Keywords:** breast cancer, biomarker, DHCR7, proliferation, mitochondrial function

## Abstract

Background: Energy metabolism has a complex intersection with pathogenesis and development of breast cancer (BC). This allows for the possibility of identifying energy-metabolism-related genes (EMRGs) as novel prognostic biomarkers for BC. 7-dehydrocholesterol reductase (DHCR7) is a key enzyme of cholesterol biosynthesis involved in many cancers, and in this paper, we investigate the effects of DHCR7 on the proliferation and mitochondrial function of BC.

Methods: EMRGs were identified from the Gene Expression Omnibus (GEO) and MSigDB databases using bioinformatics methods. Key EMRGs of BC were then identified and validated by functional enrichment analysis, interaction analysis, weighted gene co-expression network analysis (WGCNA), least absolute shrinkage and selection operator (LASSO) regression, Cox analysis, and immune infiltration. Western blot, qRT-PCR, immunohistochemistry (IHC), MTT assay, colony formation assay and flow cytometry assay were then used to analyze DHCR7 expression and its biological effects on BC cells.

Results: We identified 31 EMRGs in BC. These 31 EMRGs and related transcription factors (TFs), miRNAs, and drugs were enriched in glycerophospholipid metabolism, glycoprotein metabolic process, breast cancer, and cell cycle. Crucially, DHCR7 was a key EMRG in BC identified and validated by WGCNA, LASSO regression and receiver operating characteristic (ROC) curve analysis. High DHCR7 expression was significantly associated with tumor immune infiltration level, pathological M, and poor prognosis in BC. In addition, DHCR7 knockdown inhibited cell proliferation, induced apoptosis and affected mitochondrial function in BC cells.

Conclusions: DHCR7 was found to be a key EMRG up-regulated in BC cells. This study is the first to our knowledge to report that DHCR7 acts as an oncogene in BC, which might become a novel therapeutic target for BC patients.

## INTRODUCTION

Breast cancer (BC) ranks first in incidence and fifth in mortality among all cancers worldwide [1]. ER+, HER2+, and triple-negative breast cancer (TNBC) are the major clinical subtypes for BC, of which ER+ cancers account for approximately 70% of total cases [2]. Many risk factors contribute to BC’s high incidence rate and mortality. These factors include increased age, being female, genetics, hormones, higher alcohol consumption, obesity, and diabetes [3, 4]. Although many of the most effective diagnostic methods and targeted drugs are used for BC patients, the survival outcomes are still poor [5]. Therefore, it is critical to identify novel tumor biomarkers and therapeutic targets for BC diagnosis and treatment.

Altered energy metabolism is a hallmark of the occurrence and development of cancers [6], and previous studies have suggested that gene expression regulates energy metabolism and influences pathogenesis, microenvironment, and therapy in tumors [7]. LINC00242 has been found to be highly expressed in gastric cancer, and silencing of LINC00242 has likewise been shown to inhibit gastric cancer cells proliferation significantly by regulating cells aerobic glycolysis [8]. Furthermore, the GTPBP4-PKM2 regulatory axis contributes to cells proliferation and metastasis, which is involved in glycolysis and the TCA cycle in hepatocellular carcinoma (HCC) [9]. Researchers have also found that metabolic reprogramming and immune evasion played important roles in BC cell proliferation through regulated glucose metabolism and lipid metabolism [10]. In addition, increasing amounts of evidence has indicated that mitochondrial metabolism is an important therapy target in many cancers and that altering metabolic pathways may inhibit cancer growth, especially for TNBC [11–14]. Therefore, identification of EMRGs in BC may provide novel strategies to improve prognosis and treatment.

7-Dehydrocholesterol reductase (DHCR7) is a cholesterol epoxide hydrolase involved in fetal development and growth, tumor cell differentiation, and apoptosis [15, 16]. Gabitova et al. found that emopamyl-binding protein (EBP) in a complex with DHCR7 catalyzes the production of cholesterol epoxide hydrolase (ChEH), which is closely related to tumor cells growth [17]. DHCR7 is already recognized as a risk factor in several tumors via regulating circulating vitamin D concentration, including ovarian cancer [18], nonmelanoma skin cancer [19], and thyroid cancer [20]. In addition, DHCR7 has been shown to be an oncogene and its high expression regulates the proliferation, migration, and invasion as well as apoptosis of tumor cells, such as in bladder cancer [21] and gastric cancer [22]. Additionally, DHCR7 is a vitamin D-related gene that interacts with 25(OH)D to increase the risk of BC [23]. However, DHCR7 expression and its related molecular functions remain unclear in BC.

**Figure 1:**
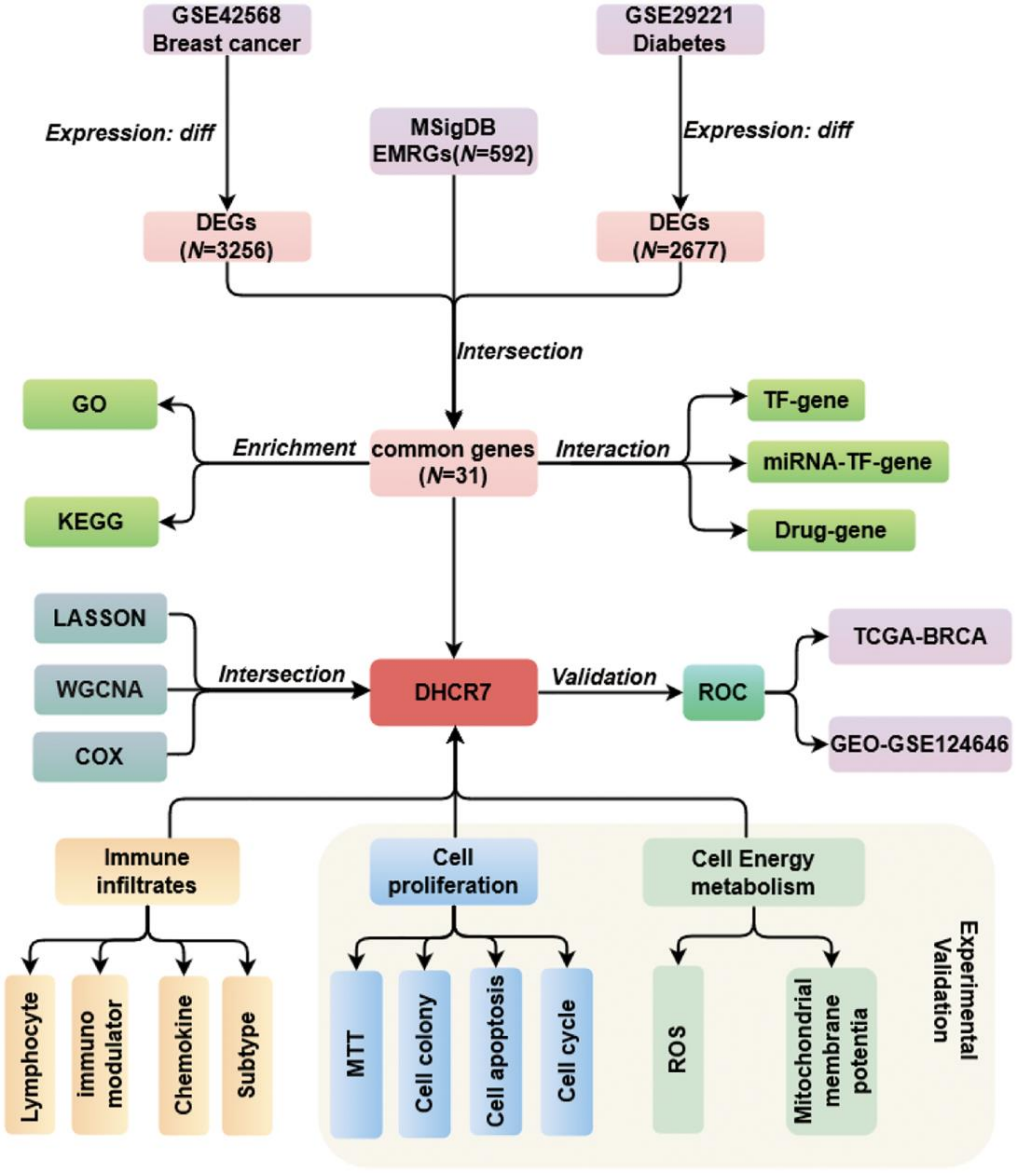
Flow diagram of overall study.

In this study, EMRGs were identified in public databases using bioinformatics methods. Gene ontology (GO) is widely used in annotating genes with such properties as biological process (BP), cellular component (CC), and molecular function (MF), and the Kyoto Encyclopedia of Genes and Genomes (KEGG) is utilized to predict signaling pathways of genes by linking genomic information with higher order functional information [24, 25]. We used both GO, KEGG and interaction analysis to reveal the potential biological function of the identified EMRGs. After integrated analysis consisting of LASSO regression, WGCNA, Cox analysis, and ROC curve analysis, DHCR7 was identified and validated as a key EMRGs in BC. Finally, we investigated the biological role of DHCR7 in BC cells proliferation and mitochondrial function. The flow diagram of this study is shown in Figure 1. This study aims to provide a novel potential therapeutic target for BC treatment.

## METHODS

### Data processing and identification of common EMRGs

Two microarray data sets (breast cancer, GSE42568; diabetes, GSE29221) were downloaded from the Gene Expression Omnibus (GEO) database (accessed in January 2023). The “limma” R package was utilized to identify the differentially expressed genes (DEGs), and |logFC| > 1.5 and *P*-value < 0.05 were used as the criteria for DEGs screening [26]. 592 energy metabolism related genes (EMRGs) were obtained from the MSigDB database (http://www.broad.mit.edu/gsea/msigdb/, accessed in January 2023) as well, and an UpSet Venn diagram was used to intersect common genes using the image GP website (https://www.bic.ac.cn/ImageGP/index.php/Home/Index/UpsetView.html, accessed in January 2023). Detailed microarray data sets information is presented in Supplementary Table 1. The DEG and EMRG lists are shown in Supplementary Table 2.

### GO and KEGG enrichment analysis of common EMRGs

In order to reveal the underlying functions and mechanisms of common EMRGs, the DAVID database (accessed in January 2023) was used to perform GO and KEGG pathway enrichment analysis of common EMRGs for annotation and visualization. The top 10 statistically significantly enriched GO and KEGG terms were identified with *P* < 0.05 as the threshold for statistical significance.

### Interaction analysis of common EMRGs, miRNAs, TFs, and drugs

We used the NetworkAnalyst website (http://www.networkanalyst.ca/faces/home.xhtml, accessed in January 2023) to obtain interaction network and KEGG pathways of common EMRGs, miRNAs, TFs, and drugs. All of our identified common EMRGs were uploaded into the NetworkAnalyst tool. After the organism (H. sapiens) and Set ID type (official Gene symbol) were chosen, the analysis of TF-gene interactions, TF-miRNA coregulatory networks, and protein-drug interactions were each performed. The KEGG database was used as function explorer for all nodes. The top 10 KEGG terms criteria were determined by the following criteria: *P* < 0.05 and node degree >5.

### Identification and validation of key EMRGs

The weighted gene co-expression network (WGCNA) R package was used to identify the key genes from breast cancer samples and the corresponding clinical data [27], and LASSO regression was performed to obtain the expression values of gene diagnosis using the “glmnet” R package (seed = 100, cross-validation (CV) = 5) [28]. Independent risk genes associated with the overall survival of breast cancer patients were determined using Cox regression. Furthermore, we also used ROC curves to evaluate the specificity and sensitivity of the EMRGs. The validation datasets were acquired from the GEO database (GSE124646) and The Cancer Genome Atlas (TCGA) database (BRCA).

### DHCR7 expression in immune and molecular subtypes of BC

Immune infiltrates are very important to the cancer initiation and progression. Hence, the TISIDB (http://cis.hku.hk/TISIDB/index.php, accessed in January 2023) was used to analyze the relationship between DHCR7 expression and immune subtype, molecular subtype, tumor-infiltrating lymphocytes (TILs), immunomodulators chemokines, and receptors, including immunoinhibitor, immunostimulator, and MHC molecule. *P*-values < 0.05 were considered to indicate statistically significant test results.

### Correlation and GSEA analysis

RNA sequencing data and clinicopathological information for BRCA were downloaded from the TCGA (https://xena.ucsc.edu, accessed in January 2023). 11 genes (BIRC5, CCNB1, CDC20, NUF2, CEP55, NDC80, MKI67, PTTG1, RRM2, TYMS, and UBE2C) related to breast cancer proliferation that had been validated by experiment were obtained from the published articles [29]. After this, correlation analysis of DHCR7 expression and tumor stage, survival analysis, proliferation related genes was performed using “ggcorrplot” R packages [30, 31]. The Kaplan-Meier plotter was used for gene survival analysis (http://kmplot.com/analysis, accessed in January 2023). Gene set enrichment analysis (GSEA) was performed using GSEA software v4.2.3 (http://www.gsea-msigdb.org/gsea/index.jsp, accessed in January 2023) [32], and once again *P*-values less than 0.05 were used to indicate statistically significant test results.

### Cell culture and siRNA transfection

The following human breast cancer cell lines were obtained from Genechem (Shanghai Genechem Co., Ltd., Shanghai, China) in this study for practical experimentation: MCF-7, MDA-MB-231, and DU4475, and we also used the breast epithelial cell line MCF-10A. MCF-7, MDA-MB-231, and DU4475 were cultured in DMEM medium (PAA Laboratories GmbH, Pasching, Austria) supplemented with 10% fetal bovine serum (FBS, PAA Laboratories GmbH), and MCF-10A was cultured in DMEM/F12 medium supplemented with 10% FBS (Procell Life Science and Technology, Wuhan, China), and incubated in a humidified 5% CO_2_ incubator at 37°C. The small interfering RNA (siRNA) to target DHCR7 was designed and synthesized from Sangon Biotech (Shanghai, China). Non-sense siRNA was used as a control, and this siRNA was transfected into the cells using Lipofectamine 2000 (Invitrogen, USA). All siRNA sequences are shown in Supplementary Table 3.

### Cell viability and colony formation assay

The MTT assay (Sigma, USA) was used to measure cell proliferation. MDA-MB-231 cells were seeded into 96-well plates (3000 cells per well). At 24, 48, and 72 h after transfection, the MTT kit was added to the 96-well plates (10 μL per well). The 96-well plates were then incubated at 37°C for 4-6 h, and the supernatant in each well was replaced with 150 μL DMSO. Finally, the absorbance was detected by a microplate reader (OD: 492 nm). Cells were then seeded into 6-well plates with 2 mL complete medium (200 cells per well) and cultured for 10–14 days. Colonies were then fixed with methanol and stained with a crystal violet solution (Sigma-Aldrich, USA) for 20 min. Finally, the colonies were photographed and counted using Quantity One software (Bio-Rad Laboratories, USA). Each assay was carried out in triplicate.

### Cell cycle and apoptosis assays

The MDA-MB-231 cells were also cultured in 6-well plates at 2×10^5^ per well in order to facilitate cell cycle and apoptosis assays. Cells were treated with siRNA transfection, washed, and fixed with 70% ethanol for 24 h. Afterwards, 100 μL Rnase A and 400 μL propidium iodide (PI) (7Sea Biotech, Shanghai, China) were added to the 6-well plates. Finally, cell cycle distribution was analyzed by flow cytometer. The cell apoptosis assay was performed by flow cytometer according to the manufacturer’s instructions for the Annexin-VFITC Apoptosis Detection Kit (Invitrogen, USA). Each measurement was performed in triplicate.

### ROS detection

For ROS detection, the MDA-MB-231 cells were seeded into 6-well plates treated with DHCR7 siRNA at 37°C in 5% CO_2_. After 48 h, the cells were harvested, washed with phosphate-buffered saline (PBS) for three times, and then stained with 10 μM DCFH-DA at 37°C for 20 min according to the directions for the ROS Assay Kit (Beyotime, China). The stained cells were then analyzed using a flow cytometer and fluorescence microscope. All experiments were performed in triplicate.

### Mitochondrial membrane potential (MMP) detection

The MDA-MB-231 cells were seeded into 6-well plates and cultured in DMEM supplemented with 10 % FBS. After transfection for 48 h, the mitochondrial transmembrane potential (ΔΨm) of the MDA-MB-231 cells was determined using a JC-1 Mitochondrial Membrane Potential Assay Kit (Yeasen, Shanghai, China). Low ΔΨm indicates green fluorescence, and high ΔΨm indicates red fluorescence. Briefly, MDA-MB-231 cells were stained with JC-1 (diluted 1:200) at 37°C for 20 min following the manufacturer’s instructions. Flow cytometer and fluorescence microscope were then used to measure the mitochondrial membrane potential of the cells. Each measurement was performed in triplicate.

### Immunohistochemistry (IHC)

Paired human breast cancer samples were collected from patients who had undergone mastectomy at Shaanxi Provincial Tumor Hospital, all samples were frozen in liquid nitrogen after operation. Both tumor and non-tumor tissues were validated via pathological examination. The study was approved by the Ethics Committee of Shaanxi Provincial People’s Hospital, and informed consent was obtained from all patients. The tissues were fixed for 24–36 h with 4% paraformaldehyde, and then embedded in paraffin. The samples were made into 5 μm sections, and then the sections were treated, deparaffinized, hydrated, subjected to antigen retrieval, also subjected to the endogenous breaking, blocked, and incubated with primary antibody overnight at 4°C prior to incubation with the secondary antibody for 2 h. A DAB kit (Sigma, USA) and hematoxylin were used to measure the sections, and the intensity of staining was evaluated by a Leica Q550 image analysis system.

### qRT-PCR

Total RNA was prepared from cell lines using TRIzol reagent (Invitrogen, USA), and the cDNA was generated using a PrimeScript RT reagent Kit (Takara, Japan). A SYBR Green PCR kit (Takara, Japan) was then used to perform Quantitative real-time PCR (qRT-PCR). The relative expression of DHCR7 and GAPDH was calculated using the 2^−ΔΔCt^ method, and the primers were designed and synthesized from Sangon Biotech (Shanghai, China). Supplementary Table 3 shows the primer sequences. PCR was performed in three parallel holes using the IQ-5 Real-Time PCR System (Bio-Rad).

### Western blot

For western blot analysis, RIPA buffer (Invitrogen, USA) was used to lyse the cell samples. Protein concentration was then determined using a BCA protein assay kit (GenStar, China). Equal amounts of protein were separated by 10% SDS PAGE gel, and subsequently transferred to a methanol-activated PVDF membrane (Millipore, USA). These membranes were blocked with 5% non-fat milk for 2 h at room temperature and then incubated overnight at 4°C with primary antibody. After washing three times with tris-buffered saline containing 0.1% Tween 20 (TBST), the membranes were incubated with secondary antibody for 2 h. The protein bands were then detected and analyzed using the Bio–Rad chemiluminescence imaging system. The antibody details were as follows: DHCR7 (PA5-48204, Invitrogen), Bcl2 (CY5032, Abways, China), CDK6 (66278-1-lg, Proteintech, China), Caspase 9 (66169-1-lg, Proteintech), β-actin (66009-1-lg, Proteintech), Goat Anti-mouse (IgG) (SA00001-1, Proteintech), and Goat Anti-Rabbit IgG (SA00001-2, Proteintech).

### Statistical analysis

Prior to analysis, all data were expressed as the mean ± SD of three independent experiments. Statistical analysis was then performed with GraphPad Prism 7.0 software and SPSS Statistics 25 (IBM). Student’s *t*-test was used for comparisons of differences between group, and one-way ANOVA was used to analyze multiple group differences. For all tests, *P* < 0.05 was used to indicate statistical significance.

## RESULTS

### Identification of 31 EMRGs in breast cancer

**Figure 2:**
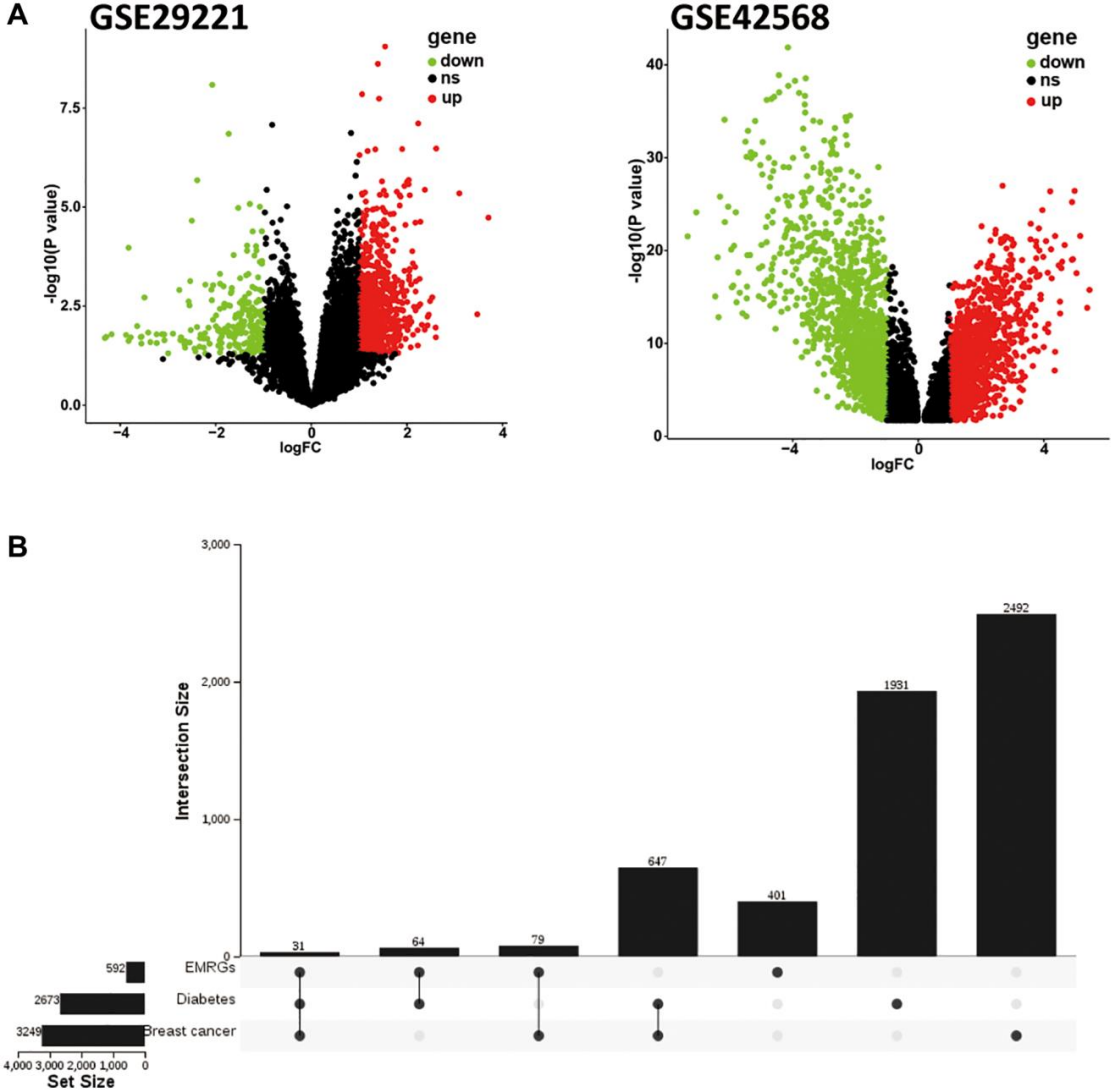
**Identification of EMRGs in BC.** (**A**) Volcano plots of DEGs in breast cancer (GSE42568) and diabetes mellitus (GSE29221). (**B**) UpSet Venn diagram showing the most common EMRGs. Black points: ns (no significant); Red points: up-regulated genes; Green points: down-regulated genes.

To identify the EMRGs in breast cancer, we analyzed RNA sequence data of BC (GSE42568) and DM (GSE29221) from the GEO database using the “limma” package in R (*P* < 0.05, |logFC| > 1.5). 3,249 DEGs were identified for BC, including 1,624 up-regulated and 1,625 down-regulated genes, whereas the DEGs identified in the DM included 2,106 up-regulated and 567 down-regulated genes (Figure 2A). After intersecting 592 EMRGs, we identified 31 common genes identified using an UpSet Venn diagram (Figure 2B).

### GO and KEGG enrichment analysis of the 31 EMRGs

**Figure 3:**
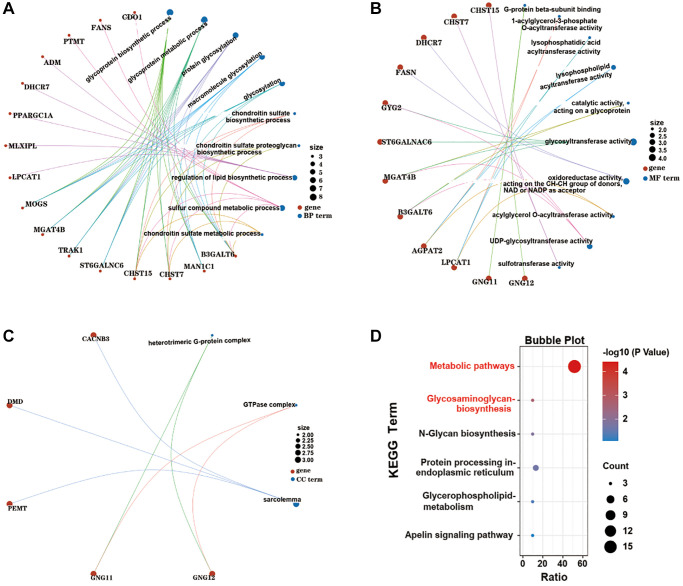
**GO and KEGG pathway enrichment analysis of the most common EMRGs.** (**A**) BP terms of the EMRGs. (**B**) MF terms of the EMRGs. (**C**) CC terms of the EMRGs. (**D**) KEGG terms of the EMRGs.

For functional and pathway enrichment analysis, the 31 EMRGs were uploaded into the DAVID database. GO functional analysis included three parts: biological process (BP), molecular function (MF) and cell component (CC), where the selection criterion was *P* < 0.05. GO BP analysis of the 31 EMRGs showed that the genes were mostly enriched in glycoprotein biosynthetic process, glycoprotein metabolic process, protein glycosylation and regulation of lipid biosynthetic process; GO MF analysis revealed that the 31 EMRGs were primarily enriched in G-protein beta-subunit binding, lysophosphatidic acid acyltransferase activity, and glycosyltransferase activity; and GO CC analysis indicated that they were significantly enriched in heterotrimeric G-protein complex, GTPase complex and sarcolemma sarcolem. More details of GO functional terms are displayed in Figure 3A–3C. Furthermore, the results of KEGG analysis are shown in Figure 3D, where the 6 top enriched pathways were identified and included metabolic pathways, glycosaminoglycan biosynthesis, N-Glycan biosynthesis, protein processing in endoplasmic reticulum, glycerophospholipid metabolism, and Apelin signaling pathway.

### Identifying functional networks of the 31 EMRGs, miRNAs, TFs and drugs

**Figure 4:**
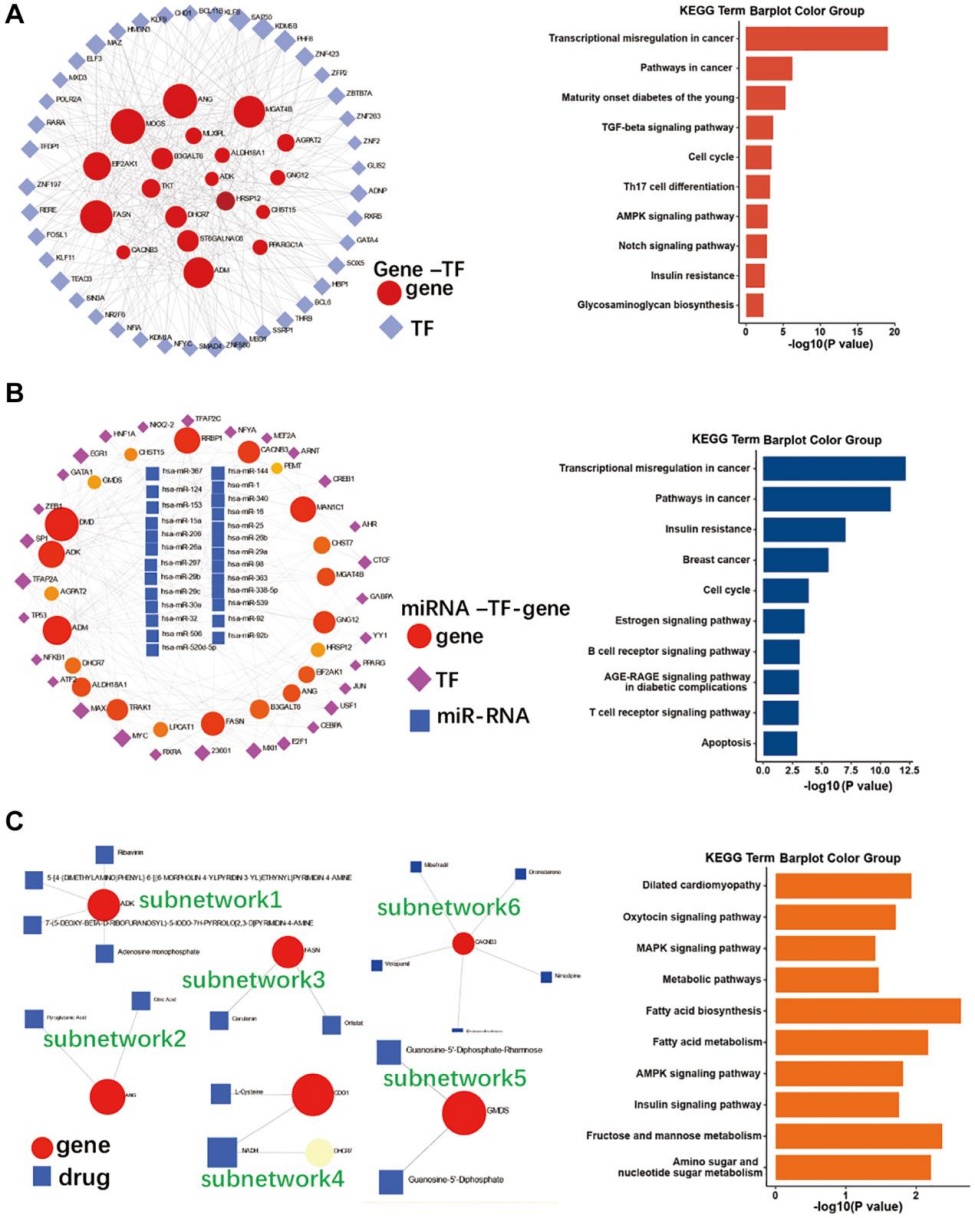
**Co-regulatory network of EMRGs, miRNAs, TFs, and drugs.** (**A**) TF-gene co-regulatory network of and its KEGG pathway enrichment. (**B**) miR-RNA-TF-gene co-regulatory network and its KEGG pathway enrichment. (**C**) Gene-drug co-regulatory network and its KEGG pathway enrichment.

To explore the functional networks of the 31 EMRGs, miRNAs, TFs and drugs, the EMRGs were imported into the NetworkAnalyst online tool. Our interaction analysis of EMRGs and TFs showed that 19 EMRGs and 41 TFs formed an interaction network, and these were mainly enriched in 10 pathways, including transcriptional misregulation in cancer, pathways in cancer, insulin resistance, AMPK signaling pathway, and cell cycle (Figure 4A). The interaction network of miRNA-TF-gene was also constructed and had 22 EMRGs, 29 TFs and 26 miRNAs enriched in 10 pathways (top5: transcriptional misregulation in cancer, pathways in cancer, insulin resistance, breast cancer, and cell cycle) (Figure 4B). As shown in Figure 4C, gene-drug interaction networks included 6 subnetworks with 7 EMRGs and 17 drugs and were significantly enriched in dilated cardiomyopathy, adrenergic signaling in cardiomyocytes, oxytocin signaling pathway, and AMPK signaling pathway.

### Identification of the key EMRGs in breast cancer

**Figure 5:**
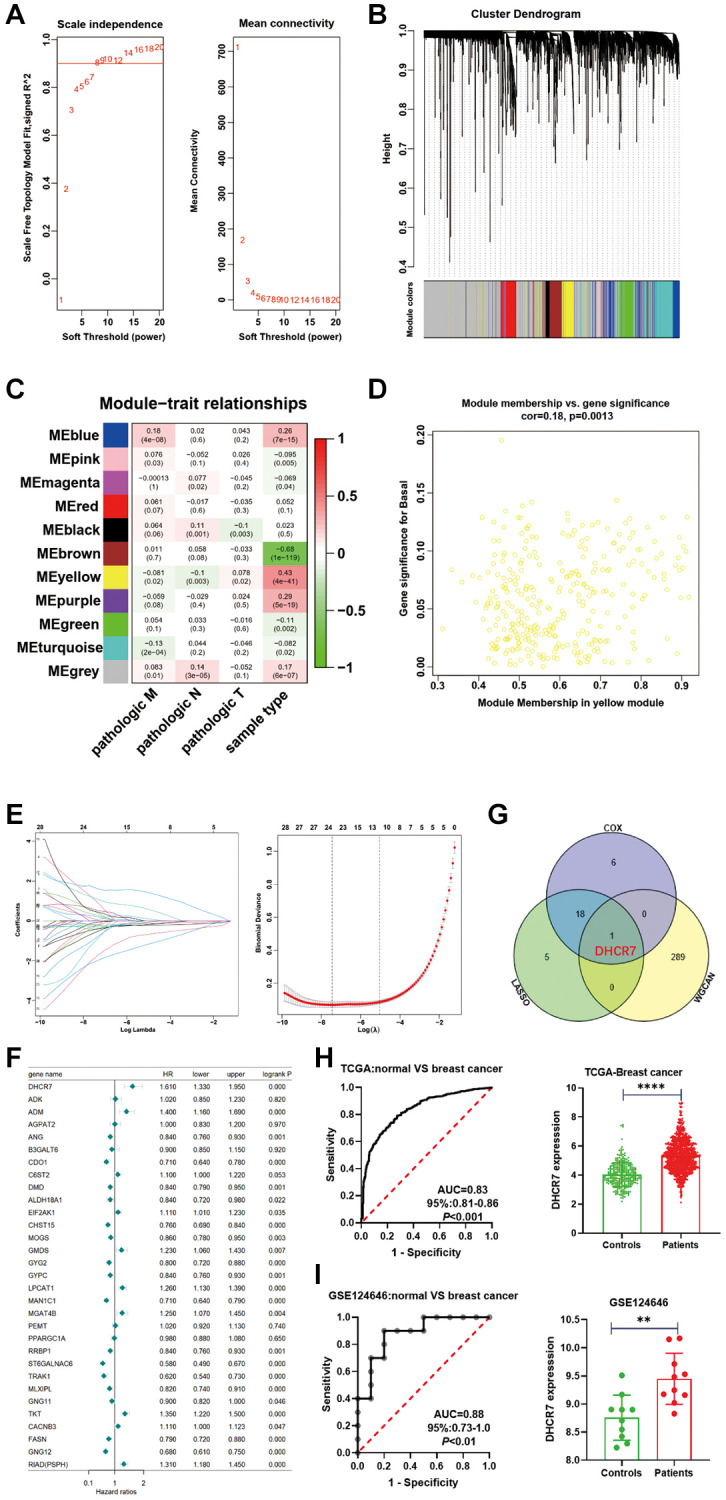
**Identification of the key EMRGs in BC samples.** (**A**) Selection of the soft threshold using a scale-free network. (**B**) Co-expression modules of BC using WGCNA. (**C**) Correlation analysis of gene modules and BC clinical traits. (**D**) Scatter plot of the yellow module. (**E**) Screening of key EMRGs by LASSO regression. (**F**) Cox regression analysis of the 31 EMRGs expression and OS of BC. (**G**) Venn diagram analysis of key EMRGs. (**H**) ROC analysis for DHCR7 expression in the TCGA dataset. (**I**) ROC analysis for DHCR7 expression in the validation dataset (GSE124646). ^**^p < 0.01; ^****^p < 0.0001.

WGCNA was used to identify the key gene modules of BC. As shown in Figure 5A, we obtained a higher average degree of network connectivity using a soft threshold (β = 8). 11 gene co-expression modules were identified, including MEblue, MEpink, MEmagenta, MEred, MEblack, MEbrown, MEyellow, MEpurple, MEgreen, MEturquoise, and ME gray, and these had a significant correlation with both pathological (TNM) and sample types (Figure 5B, 5C). Correlation analysis results show that the 290 MEyellow genes were most related to sample types (Figure 5D). 24 candidate genes were also identified from the 31 EMRGs using LASSO regression, as shown in Figure 5E. The Cox analysis was then performed to validate the correlation between EMRGs expression and overall survival (OS). Forest plots indicated that 8 EMRGs were considered a risk factor for BC, including DHCR7, ADM, EIF2AK1, GMDS, LPCAT1, MGAT4B, TKT, and RIAD (HR > 1, *P* < 0.05), and 14 EMRGs acted as a protective prognostic factor for BC, including ALDH18A1, ANG, CDO1, DMD, CHST15, GYG2, GYPC, MAN1C1, RRBP1, ST6GALNAC6, TRAK1, MLXIPL, FASN, and GNG12 (Figure 5F). The Venn diagram revealed that DHCR7 was a key EMRG after intersecting the results of WGCNA, LASSO regression, and Cox analysis (Figure 5G). Furthermore, we used an ROC curve to evaluate and validate the diagnostic value of DHCR7 and found that the area under the curve (AUC) was 0.83 (95% CI = 0.81–0.86, *P* < 0.001) in the TCGA-BRCA dataset and that DHCR7 was up-regulated in BC (Figure 5H). The GSE124646 was also used as an external dataset to validate this result, which also yielded a high DHCR7 expression and AUC value (95% CI = 0.73–1.0, *P* < 0.01) for BC (Figure 5I). The above results show that DHCR7 is a key EMRGs in BC.

### Association of DHCR7 expression with immune characteristics in breast cancer

**Figure 6:**
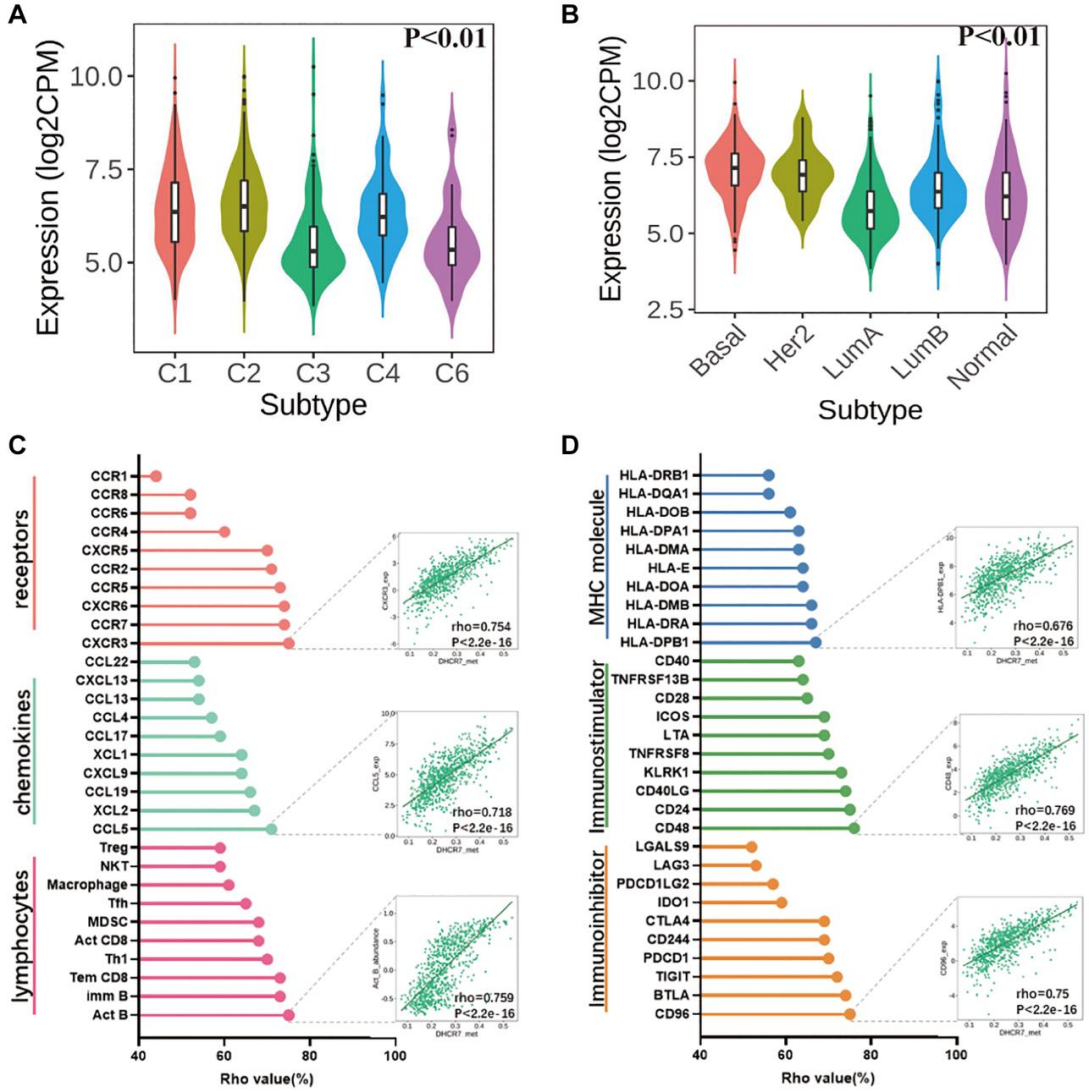
**The correlation between DHCR7 expression and BC immune characteristics.** (**A**) DHCR7 expression correlations with 5 immune subtypes in BC. (**B**) DHCR7 expression was related to the molecular subtypes of BC. (**C**) DHCR7 expression correlations with lymphocytes chemokines and receptors. (**D**) Correlations between DHCR7 expression and 3 kinds of immunomodulators.

Tumor immune infiltration has a profound effect on survival and immunotherapy efficacy for cancer patients. For this reason, we used the TISIDB database to investigate the correlation of DHCR7 expression with immunomodulators, chemokine, immune, and molecular subtypes. We found that the DHCR7 expression was related to 5 immune subtypes in BC, including wound healing (C1), IFN-γ dominant (C2), inflammatory (C3), lymphocyte depletion (C4) and TGF-β dominant (C6) (Figure 6A). DHCR7 expression was also significantly correlated with molecular subtypes of BC (Figure 6B). In Figure 6C, we can see that DHCR7 expression was positively correlated to lymphocytes, especially in Act B cells (rho = 0.759, *P* < 2.2e-16). In addition, DHCR7 expression had a significantly positive correlation with chemokines (CCL5, rho = 0.718, *P* < 2.2e-16) and their receptors (CXCR3, rho = 0.754, *P* < 2.2e-16). We also investigated correlations between three kinds of immunomodulators and DHCR7 expression and found that DHCR7 expression was significantly related to immunoinhibitor (CD96, rho = 0.75, *P* < 2.2e-16), immunostimulator (CD48, rho = 0.769, *P* < 2.2e-16), and MHC molecules (HLA-DPB1, rho = 0.676, *P* < 2.2e-16), as shown in Figure 6D.

### DHCR7 is up-regulated in BC tissues and connected with OS

**Figure 7:**
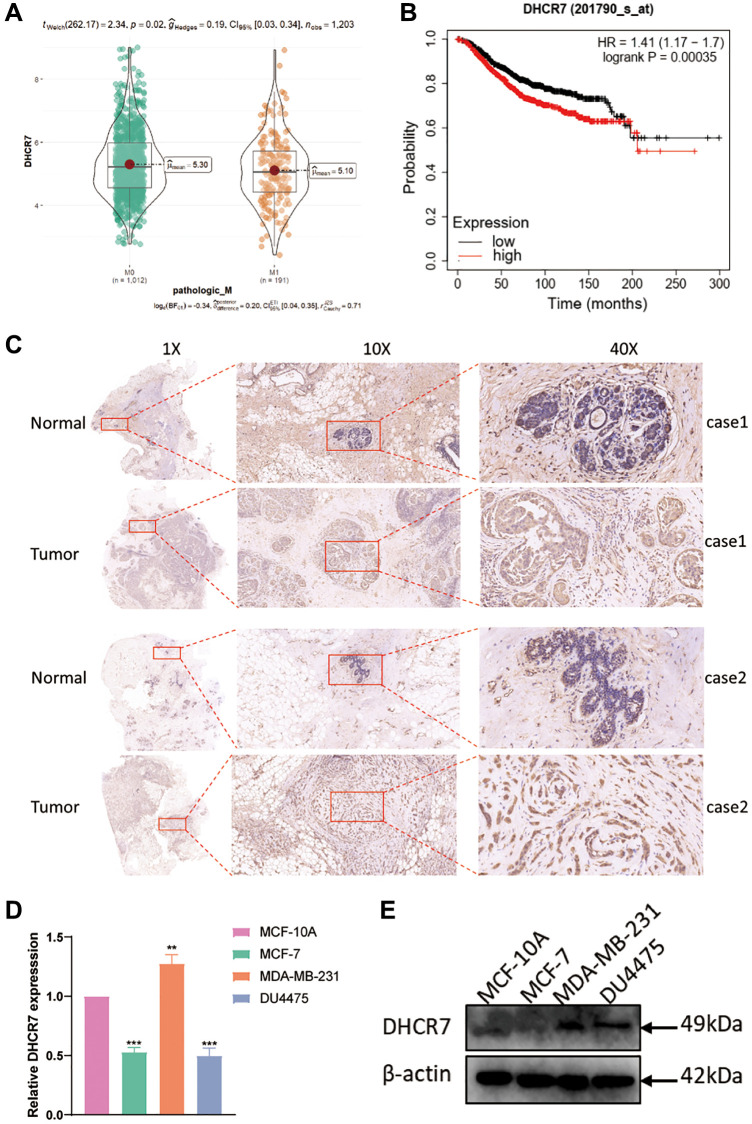
**Analysis of DHCR7 expression and survival in patients with BC.** (**A**) Correlation analysis between DHCR7 expression and pathological M. (**B**) Higher DHCR7 expression induced poorer survival of BC patients. (**C**) IHC detected DHCR7 expression in BC tissues and adjacent normal tissues. (**D**, **E**) qRT-PCR and western blot examined DHCR7 expression in cell lines. ^**^p < 0.01; ^***^p < 0.001.

Based on our TCGA database analysis, we found that DHCR7 expression was significantly correlated with the pathological M of BC patients (*P* = 0.02) (Figure 7A). Survival analysis also showed that higher DHCR7 expression resulted in the poorer probability of survival (HR = 1.4, *P* = 0.00035) (Figure 7B). By using IHC, we further found that the protein level of DHCR7 was up-regulated in human BC tissues compared to adjacent normal tissues (Figure 7C). To investigate DHCR7 expression in BC further, we then analyzed DHCR7 expression in our cell lines. The results of our qRT-PCR testing showed that DHCR7 expression was significantly higher in MDA-MB-231 cells compared to MCF-10A cells, but lower in MCF-7 and DU4475 cells (Figure 7D). In addition, western blot showed that DHCR7 protein level was up-regulated in MDA-MB-231, DU4475 cells, and MCF-7 cells compared to MCF-10A cells (Figure 7E). Therefore, MDA-MB-231 cells were used for further experiments.

### DHCR7 knockdown inhibited proliferation and induced apoptosis in MDA-MB-231 cells

**Figure 8:**
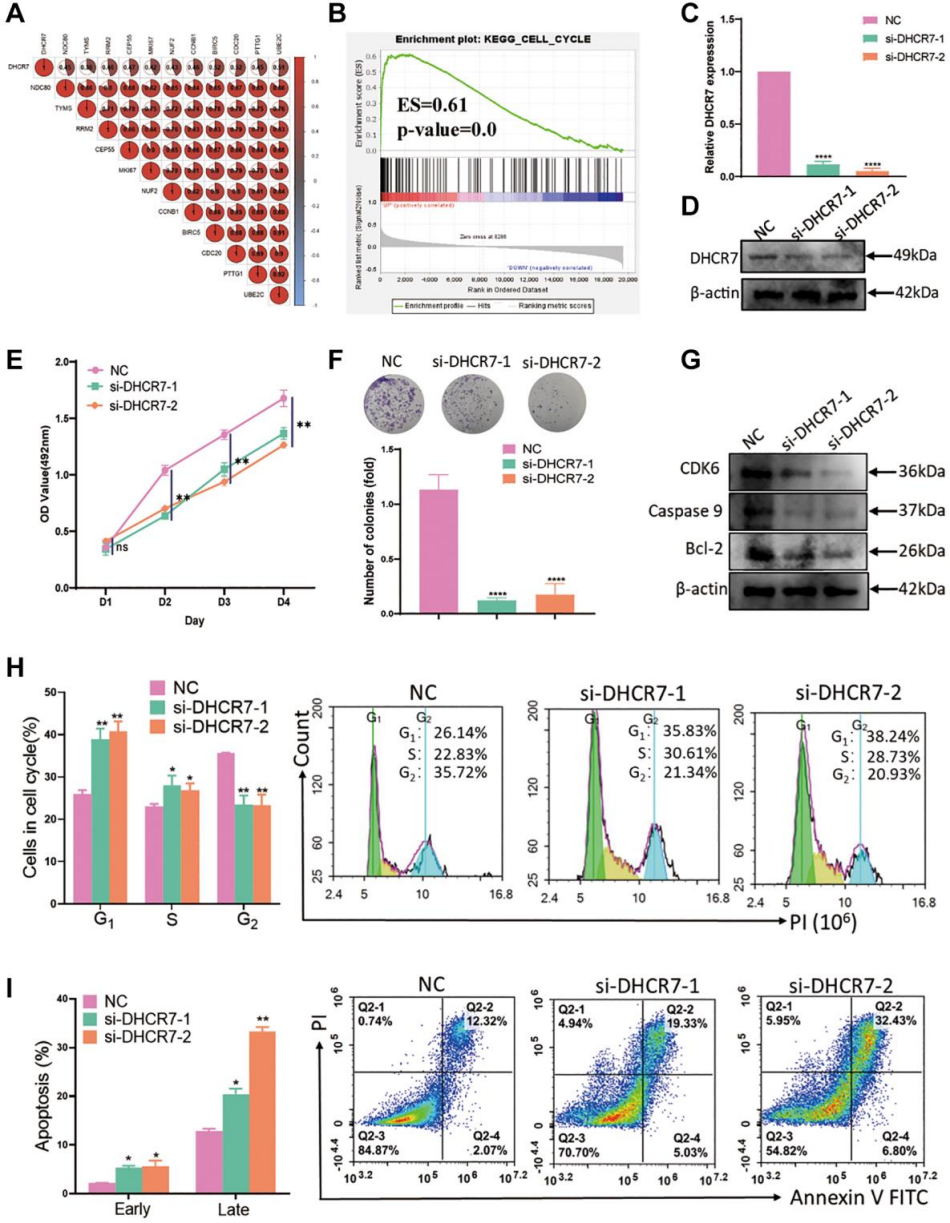
**DHCR7 knockdown inhibited proliferation and induced apoptosis of MDA-MB-231 cells.** (**A**) Correlation between DHCR7 expression and 11 genes related to breast cancer proliferation. (**B**) GSEA analysis of DHCR7 expression in BC. (**C**, **D**) qRT-PCR and western blot determined the efficiency of DHCR7 knockdown in MDA-MB-231 cells. (**E**) MTT assay investigated the MDA-MB-231 cells viability with DHCR7 knockdown. (**F**) The effect of DHCR7 knockdown on colony formation of MDA-MB-231 cells. (**G**) Western blot detected the expression of CDK6, caspase9, and Bcl2. (**H**) Flow cytometry assay tested the cell cycle of MDA-MB-231 cells with DHCR7 siRNA. (**I**) DHCR7 knockdown remarkably induced apoptosis of MDA-MB-231 cells. Abbreviation: NC: negative control. ^*^p < 0.05; ^**^p < 0.01; ^***^p < 0.001; ^****^p < 0.0001.

In order to investigate the effect of DHCR7 expression on cell proliferation, we performed GSEA and Pearson’s analysis. Pearson’s analysis showed that DHCR7 expression was markedly correlated with 11 genes related to breast cancer proliferation, including BIRC5, CCNB1, CDC20, NUF2, CEP55, NDC80, MKI67, PTTG1, RRM2, TYMS, and UBE2C (Figure 8A). GSEA analysis additionally revealed that high DHCR7 expression was mainly enriched in cell cycle (ES = 0.61, *P* < 0.01) in BC (Figure 8B). After siRNA was transfected into the MDA-MB-231 cells, qRT-PCR and western blot were used to test the efficacy of DHCR7 knockdown. As shown in Figure 8C, 8D, DHCR7 expression was successfully decreased in MDA-MB-231 cells at the level of mRNA and protein. MTT and colony formation assay further revealed that DHCR7 knockdown significantly suppressed MDA-MB-231 cells proliferation (Figure 8E, 8F). Furthermore, cell cycle assay showed that DHCR7 knockdown resulted G0/G1 phase arrest of MDA-MB-231 (Figure 8H). In addition, DHCR7 knockdown increased both early and late apoptosis in the MDA-MB-231 cells (Figure 8I), and the expression levels of CDK6, caspase9, and Bcl2 were decreased in MDA-MB-231 cells transfected with DHCR7 siRNA (Figure 8G). Based on the above results, we conclude that DHCR7 knockdown inhibited MDA-MB-231 cells proliferation and induced apoptosis.

### The effects of DHCR7 knockdown on ROS generation and MMP levels of MDA-MB-231 cells

**Figure 9:**
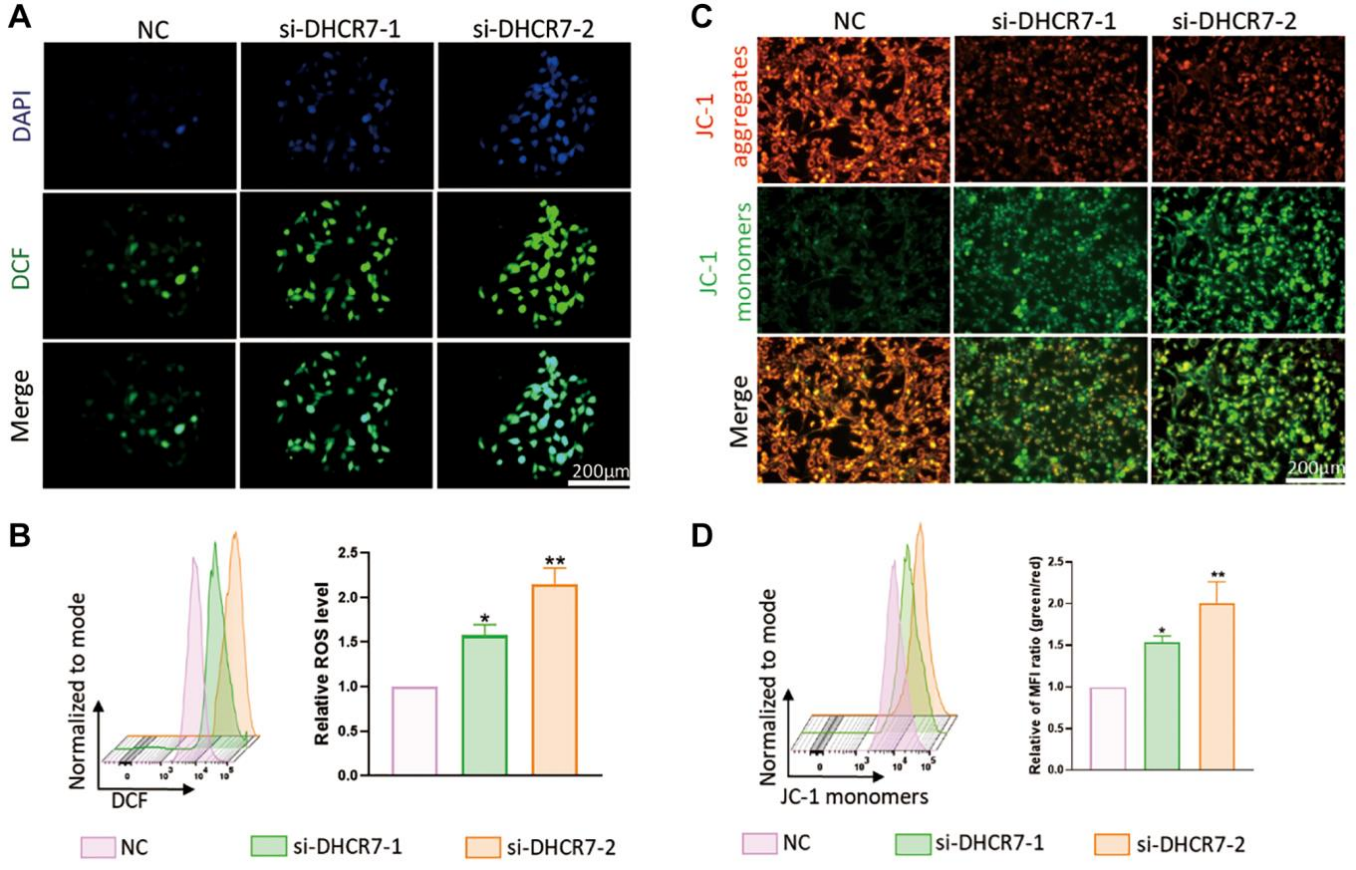
**DHCR7 knockdown influenced mitochondrial function of MDA-MB-231 cells.** (**A**, **B**) MDA-MB-231 cells were treated with DHCR7 siRNA. ROS production was detected by fluorescence microscope and flow cytometer. (**C**, **D**) The level of MMP was detected by JC-1 staining using a fluorescence microscope and flow cytometer. ^*^p < 0.05; ^**^p < 0.01.

We further detected the impact of DHCR7 knockdown in MDA-MB-231 cells on mitochondrial membrane potential (MMP) and ROS generation and found that DHCR7 knockdown induced high levels of ROS in the MDA-MB-231 cells, as evidenced by both fluorescence microscope and flow cytometry assays (Figure 9A, 9B). As shown in Figure 9C, MMP was significantly decreased in the MDA-MB-231 cells with DHCR7 knockdown, which was consistent with the MMP quantitative results using flow cytometry assays (Figure 9D). These findings suggested that DHCR7 knockdown affected the energy metabolism of MDA-MB-231 cells by adjusting the ROS and MMP.

## DISCUSSION

The occurrence of BC is a complex biological process that involves multiple factors. In recent years, with the rapid development of medical technology, many therapeutic and diagnostic targets have been applied in clinical practice for the early diagnosis or treatment of BC. However, its high incidence and mortality still remains a huge challenge for clinicians. Cellular energy metabolism has attracted more and more attention as a potential biomarker for cancers diagnosis and treatment in several tumors [7, 8, 19, 20], and with this in mind in our current study, we integrated bioinformatics methods and experiments to identify EMRGs and validated their impact on cellular biological functions in BC cells.

Oxidative stress, high blood glucose, and obesity are the main metabolic characteristics of type 2 diabetes, and they are also all well-known risk factors for BC patients [33]. In this paper, 31 common EMRGs were identified via intersection of DEGs and EMRGs between patients with BC or type 2 diabetes and healthy controls. We further analyzed the functional and pathway enrichment of these common EMRGs in order to screen key pathomechanisms. GO analysis results indicated that the 31 EMRGs were mainly enriched in glycoprotein metabolic process, regulation of lipid biosynthetic process, and glycosyltransferase activity, among others. Alpha-1-acid glycoprotein (AGP) is a key glycoprotein in the glycoprotein metabolic process, and researchers have found that AGP serves as a biomarker for BC and that AGP knockdown can inhibit the production of the inflammatory factors interleukin (IL)-1β, IL-8, and tumor necrosis factor-α in BC cells [34]. KEGG enrichment analysis further revealed that 31 EMRGs were also significantly associated with metabolic pathways, glycosaminoglycan biosynthesis, glycerophospholipid metabolism, Apelin signaling pathway, and several others. Gourgue et al. reported that blocking the Apelin inhibits TNBC growth during obesity by changing the tumour microenvironment and via the apelinergic system interference, which makes it a potential treatment strategy for patients with BC and obesity [35].

Furthermore, functional networks of the 31 EMRGs, miRNAs, TFs and drugs were constructed using the NetworkAnalyst online tool. The results for the gene-TF co-regulatory network showed that 41 TFs regulated the EMRGs and were mainly involved in transcriptional misregulation in cancer, pathways in cancer, and cell cycle. Similarly, the miRNA-TF-gene and gene-drug interaction networks were significantly enriched in transcriptional misregulation in cancer, pathways in cancer, breast cancer, cell cycle, and AMPK signaling pathway. Related studies have demonstrated that Lysine demethylase 5B (KDM5B) regulates cell proliferation and migration through AMPK-mediated lipid metabolism reprogramming in BC [36]. Qin et al. reported that miR-99a-5p targeted CDC25A affected BC cell proliferation, invasion, and apoptosis by suppressing the cell cycle pathway [37]. Altogether, the biological functions of the 31 EMRGs play important roles in the development of BC. Therefore, further exploration and confirmation of key EMRGs is still needed for BC.

We also used WGCNA to identify key functional modules related to the clinical traits used in many diseases to obtain biomarkers, such as cancers [38, 39] and neurologic diseases [40, 41]. Using WGCNA analysis assays, 290 genes were identified based on TCGA-BRCA datasets and were most related to BC sample types. We then used LASSO regression and Cox analysis to screen key EMRGs related the prognostic risk in BC. After intersecting all results, DHCR7 was identified a key EMRGs in BC. In order to test DHCR7’s diagnostic value further, we next conducted ROC curve analysis. In the TCGA-BRCA dataset, the AUC of DHCR7 was 0.83, indicating that the intersection scheme had an excellent predictive capacity. We also investigated the diagnostic value of DHCR7 in the GSE124646 dataset as validation cohorts, and the results were consistent, as have been the results of previous studies. These studies have noted that DHCR7 is a vitamin D–related genes that interacts with 25(OH)D and is a risk factor for BC incidence [42]. Combining our findings with previous studies, we see that it is important to investigate the biological function of DHCR7 in BC.

Even further investigations were required to explore the relationship between DHCR7 expression and tumors cell immune infiltration. Previous studies have shown that investigating the relevant immune subsets is beneficial to the prognosis and immunotherapy of BC patients [43]. In our study five immune subtypes of BC were found to be significantly associated with the DHCR7 expression. Moreover, DHCR7 expression also showed significant differences between BC molecular subtypes. In addition, DHCR7 expression was related to tumor-infiltrating lymphocytes (TILs), such as Act B cells, imm B cells and Tem CD8 cells. Studies have found TILs to be an effective prognostic biomarker in some BC subtypes and to be related to therapeutic outcomes and prognosis as well [43, 44]. We also examined whether chemokines (or receptors) might be regulated by DHCR7 in BC and found that DHCR7 expression was positively related to the CCL5, CCR5, and CXCR3. Previous studies have reported that CCL5 expression can affect BC metastasis and prognosis via CCR5 regulation of the Treg/CD4+CCR5+ cell ratio in BC patients [45]. At present, tumor-related immunomodulators have become an important method for BC treatment, and in this study, we found that DHCR7 expression is significantly correlated with the immunoinhibitor, immunostimulators, and MHC molecules, including CD96, CD48, and HLA-DPB1. Li et al. demonstrated that CD96 expression is correlated with poor long-term prognosis of BC patients, and targeting CD96 is another potential therapeutic strategy for BC [46]. Based on our results, DHCR7 may be an important new immunotherapy target for BC patients.

Up to now, few studies have examined the exact effect of DHCR7 on the proliferation and energy metabolism of BC cells. Our results revealed that DHCR7 expression is significantly associated with the pathological M and poor survival of BC. Furthermore, DHCR7 expression was remarkably higher in BC tissues and cell lines as measured by qRT-PCR, western blot, and IHC. The above results indicate that DHCR7 play important roles in tumor progression and serves as prognostic biomarkers for BC. However, the sample size of this study is relatively small, and larger studies are urgently needed to detail the specific relationships that may support this conclusion. Combined with previous research, we conclude that DHCR7 is an oncogene and risk factor in tumorigenesis [18, 19, 20, 23]. Therefore, it is very important to explore the biological function of DHCR7 in the BC and whether it can become a novel therapeutic target and diagnostic biomarkers for BC patients.

A growing body of evidence shows that proliferation markers are useful clinical biomarkers for BC molecular subtype classification, prognosis, diagnosis, and therapy [47, 48]. Nielsen et al. reported that 11 genes related to BC proliferation that were tested experimentally [29]. Our research showed that DHCR7 expression was positively correlated with 11 BC proliferation markers that were mainly enriched in cell cycle. Both of these results indicate that DHCR7 expression is related to the cell proliferation and may be a proliferation marker for BC. To reveal the effects of DHCR7 expression in BC proliferation even further, we also performed MTT assay, colony formation assay, and flow cytometry assay in the present study. We found that DHCR7 knockdown suppressed cell proliferation and induced apoptosis and decreased the cell cycle and apoptosis related gene expression involved in tumor carcinogenesis and proliferation, especially the CDK6, caspase9, and Bcl2 in MDA-MB-231 cells, which suggests that DHCR7 is a key oncogene in BC proliferation. This is consistent with the results of our bioinformatics analysis, but the underlying mechanism by which DHCR7 promotes the proliferation of BC still requires further study.

Energy metabolism is an important biological process required to maintain cell proliferation and is also involved in changing ROS and MMP. Previous studies have shown that leptin promotes BC cell growth, invasiveness, processes, and worsens prognosis by regulating energy metabolism [49]. Moreover, according to one recent report, inhibiting HMGB3 modulates autophagy and induces apoptosis by regulating ROS accumulation and decreasing MMP in BC cells [50]. In our study we elucidated the effect of DHCR7 on the ROS and MMP levels of BC cells; we found that DHCR7 knockdown increased ROS levels and decreased MMP in MDA-MB-231 cells, indicating that DHCR7 is a candidate oncogene in BC, which is consistent with the results of previous studies. To the best of our knowledge, this is the first time that DHCR7 expression has been shown to participate in the regulation of ROS and MMP in BC. More evidence is needed to validate these results, and if validated these results may one-day support the development of a targeted treatment for BC based on high DHCR7 expression levels.

## CONCLUSIONS

In conclusion, our findings indicate that DHCR7 is a key EMRG in BC. Higher DHCR7 expression is significantly correlated with poorer prognosis, immune infiltration and proliferation in BC. DHCR7 promotes cell proliferation and regulates the mitochondrial function of BC cells. These findings suggested that DHCR7 may serve as a potential diagnostic biomarker and immunotherapy target for BC patients.

## Supplementary Materials

Supplementary Tables 1 and 3

Supplementary Table 2
