# Supplementary material for: Identification and validation of DHCR7 as a diagnostic biomarker involved in the proliferation and mitochondrial function of breast cancer
Source: Aging (Albany NY). 2024 Mar 22;16(7):5967–86. doi: 10.18632/aging.205683 (PMC11042931; doi:10.18632/aging.205683)
Supplement: Supplementary Tables 1 and 3 [file aging-16-205683-s001.pdf]

SUPPLEMENTARY TABLES

Supplementary Table 1. Information of GEO and TCGA datasets.

GSE number	Platform	Control	BC (DM)	Samples
GSE42568	GPL570	17	104	breast tissue
GSE29221	GPL6947	12	12	skeletal muscle tissue
GSE124646	GPL96	10	10	breast tissue
TCGA_GTEx (BRCA)	Illumina	1099	292	breast tissue

Abbreviations: BC: breast cancer; DM: diabetes mellitus.

Supplementary Table 3. Primer and siRNA sequence.

Name	Sequence
DHCR7- Forward	CTCGGATCGGGAAGTGGTTTGAC
DHCR7- Reverse	GCCTTGCCTCCGTGTTCTCTTC
GAPDH- Forward	CAGGAGGCATTGCTGATGAT
GAPDH- Reverse	GAAGGCTGGGGCTCATTT
Negative Control siRNA sense	UUCUCCGAACGUGUCACGUTT
Negative Control siRNA antisense	ACGUGACACGUUCGGAGAATT
DHCR7 siRNA1 sense	CGGGAAGUGGUUUGACUUCAATT
DHCR7 siRNA1 antisense	UUGAAGUCAAACCACUUCCCGTT
DHCR7 siRNA2 sense	GCCUUAUCUUUACACGCUGCATT
DHCR7 siRNA2 antisense	UGCAGCGUGUAAAGAUAAGGCTT
